# *Rhizobium* determinants of rhizosphere persistence and root colonization

**DOI:** 10.1093/ismejo/wrae072

**Published:** 2024-05-01

**Authors:** Hayley E Knights, Vinoy K Ramachandran, Beatriz Jorrin, Raphael Ledermann, Jack D Parsons, Samuel T N Aroney, Philip S Poole

**Affiliations:** Department of Biology, University of Oxford, Oxford OX1 3RB, United Kingdom; Department of Biology, University of Oxford, Oxford OX1 3RB, United Kingdom; Department of Biology, University of Oxford, Oxford OX1 3RB, United Kingdom; Department of Biology, University of Oxford, Oxford OX1 3RB, United Kingdom; Department of Biology, University of Oxford, Oxford OX1 3RB, United Kingdom; Department of Biology, University of Oxford, Oxford OX1 3RB, United Kingdom; Department of Biology, University of Oxford, Oxford OX1 3RB, United Kingdom

**Keywords:** Rhizobium, legume, symbiosis, rhizosphere, colonization, RB-TnSeq

## Abstract

Bacterial persistence in the rhizosphere and colonization of root niches are critical for the establishment of many beneficial plant–bacteria interactions including those between *Rhizobium leguminosarum* and its host legumes. Despite this, most studies on *R. leguminosarum* have focused on its symbiotic lifestyle as an endosymbiont in root nodules. Here, we use random barcode transposon sequencing to assay gene contributions of *R. leguminosarum* during competitive growth in the rhizosphere and colonization of various plant species. This facilitated the identification of 189 genes commonly required for growth in diverse plant rhizospheres, mutation of 111 of which also affected subsequent root colonization (rhizosphere progressive), and a further 119 genes necessary for colonization. Common determinants reveal a need to synthesize essential compounds (amino acids, ribonucleotides, and cofactors), adapt metabolic function, respond to external stimuli, and withstand various stresses (such as changes in osmolarity). Additionally, chemotaxis and flagella-mediated motility are prerequisites for root colonization. Many genes showed plant-specific dependencies highlighting significant adaptation to different plant species. This work provides a greater understanding of factors promoting rhizosphere fitness and root colonization in plant-beneficial bacteria, facilitating their exploitation for agricultural benefit.

## Introduction

*Rhizobium*–legume symbioses are among the best characterized plant–bacteria interactions due to their potential to alleviate our reliance on synthetic nitrogen fertilizers. Rhizobia infect legume root nodules and fix atmospheric di-nitrogen (N_2_) into ammonia for plant utilization in return for carbon [1]. To establish symbiosis, rhizobia must undergo several lifestyle changes from saprophytic free-living bacteria in soil, to colonization of the rhizosphere (soil–root interface) and roots, followed by progression along infection threads, and finally differentiation into N_2_-fixing bacteroids. Despite this, most studies on rhizobia have focused on its symbiotic lifestyle as an endosymbiont in root nodules and therefore initial stages of rhizosphere growth and root colonization remain relatively under characterized.

Identifying factors that drive competitive nodulation of legumes by their host symbionts is of great interest because not all symbionts are equal in their biological nitrogen fixation capabilities, often resulting in suboptimal crop yields in domesticated legume species [2]. Although variation in the structure of plant-derived flavonoids and bacterial-derived Nod factors acts as checkpoints to ensure infection only proceeds with compatible symbionts, several studies suggest that selection among compatible symbionts, in part, occurs during competitive growth in the rhizosphere and root colonization [2–7]. The ability of rhizobia to catabolize carbon sources present in legume root exudates for example is required for competitive nodulation [5, 8–10]. Additionally, mutation of genes required for chemotaxis-mediated motility renders rhizobia deficient in competitive root colonization and nodule infection [11, 12].

The multifactorial nature of bacterial growth in plant rhizospheres and root colonization means that transposon insertion sequencing techniques provide a unique opportunity to study gene function at the whole genome level [7, 13–15]. We recently utilized insertion sequencing (INSeq) to identify bacterial genes important in the *Rhizobium*–legume symbiosis at multiple stages of its development [7]. This revealed that although only 27 genes are assigned roles in the organization and regulation of N_2_ fixation, 603 genetic regions were found to be required for the competitive ability to form a successful N_2-_fixing symbiosis. Of these, 146 were important for growth in the rhizosphere through to N_2_-fixing bacteroids and a further 33 from root colonization. Thus, highlighting that competition in the rhizosphere and subsequent root colonization is critical for the competitive establishment of *Rhizobium*–legume symbioses. Plants directly influence bacterial colonization of root niches, with analysis of root microbiome composition revealing variation in microbial structure among plant species and even among genotypes within a given species [16, 17]. This is primarily driven through modulation of plant root exudate composition or mounting of an innate immune response [17–19].

In the context of *Rhizobium*–legume symbioses, certain symbiont genotypes have been shown to be differentially selected by specific plant genera. *Rhizobium leguminosarum* bv. *viciae* (*Rlv*) for example can form N_2_-fixing symbioses with members of the *Fabeae* legume tribe, which consists of *Pisum*, *Lens*, *Lathyrus*, and *Vicia* species [20], but different *Fabeae* members demonstrate a preference for specific *Rlv* genotypes present in the soil population suggesting host-specific variation in competitiveness to form nodules among *Rlv* genotypes [4, 21, 22]. This altered competitiveness to form nodules is independent of their biological nitrogen fixation ability and may, at least in part, be due to altered competitiveness for growth in the rhizosphere or colonization among host legumes [2, 23, 24].

In this study, we develop random-barcode transposon-site sequencing (RB-TnSeq) in *R. leguminosarum* bv. *viciae* 3841 (Rlv3841) and utilize it to assess the genetic requirements for competitive growth in the rhizosphere and colonization of three host legumes from the *Fabeae* tribe: pea, lentil, and *Lathyrus*, as well as the non-host legume alfalfa (*Trifolieae* tribe) and a non-legume barley [25]. This has allowed us to identify a core set of 189 genes required for growth in the rhizosphere of diverse plants species, 111 of which are also required for subsequent root colonization (rhizosphere-progressive), and a further 119 genes necessary for root colonization. In addition, we identify numerous genes predicted to play plant-specific roles in these processes.

## Materials and methods

### Bacterial strains and growth conditions

Bacterial strains and plasmids are described in SI Appendix, Tables 1 and 2, respectively. *Escherichia coli* was cultured on Luria-Bertani (LB) medium and incubated at 37°C [26]. *R. leguminosarum* was cultured on Tryptone-Yeast (TY) or TY+ (SI Appendix, Table 3) and incubated at 28°C [27]. Antibiotics were added at the following concentrations (μg ml^−1^): ampicillin (Amp) 100; neomycin (Neo) 50; kanamycin (Kan) 20; nitrofurantoin (Nitro) 20; and streptomycin (Str) 500.

### RB-TnSeq library construction and sequencing

To adapt the mariner transposon delivery vector pSAM_Rl for RB-TnSeq, an oligonucleotide pool containing up to 10^13^ random 20-nuclotide barcodes flanked by universal primer binding sites was obtained from Eurofins genomics UK. Oligonucleotide and primer sequences are detailed in SI Appendix, Table 4. Barcodes were amplifed by polymerase chain reaction (PCR) using primers oxp3407 and oxp3408 such that extensions required for HiFi assembly (New England Biolabs, Ipswich, MA) directly into XhoI digested pSAM_Rl were introduced. Following HiFi assembly, modified pSAM_Rl vectors were electroporated into TransforMax EC100D pir-116 electrocompetent cells (Lucigen Simply Genomics, UK), diluted in LB supplemented with Kan + Amp, and grown overnight. Glycerol was added to 15% and 1 ml aliquots stored at −80°C. The modified pSAM_Rl vector pool was mobilized into Rlv3841 via triparental conjugation with a helper *E. coli* strain carrying plasmid pRK2013. Donor, recipient, and helper strains were pooled in a 2:2:1 ratio, pelleted via centrifugation, resuspended in 30 μl TY+, spotted onto a nitrocellulose filter placed on a TY+ agar plate and incubated at 28°C. After 24 h, the mating spot was resuspended in 1 ml TY+ supplemented with 15% glycerol and stored at −80°C. The final pool of barcoded Rlv3841 transposon mutants was generated by plating mutants on TY+ agar supplemented with Neo + Nitro. Following incubation, colonies were resuspended in TY+ supplemented with Neo + Nitro, diluted to an OD_600_ = 0.1, and grown to a final OD_600_ = 1.0. Glycerol was added to a final concentration of 20% and 1 ml aliquots were stored at -80°C. Transposon insertion sites were sequenced following the INSeq library preparation protocol previously described [28]. Reliable mutants, in which a unique barcode reliably maps to a genomic location, were identified following the previously described RB-TnSeq pipeline [25]. Any reads that did not strictly map to a TA site were computationally removed from the MapTnSeq output file.

### Plant growth, bacterial inoculation, and retrieval

Plants were grown and inoculated as previously described [7]. Briefly, seeds were sterilized (SI Appendix) and sown in 100 ml boiling tubes containing vermiculite supplemented with nitrogen and carbon-free rooting solution. For RB-TnSeq screens, an aliquot of the barcoded transposon mutant library was thawed and 10^5^ cfu inoculated per seedling at 7 days postgermination. The remainder was pelleted and stored at −20°C for DNA extraction (Time0). Bacteria were retrieved from the rhizosphere and root as previously described [7], immediately pelleted and stored at −20°C.

### RB-TnSeq mutant screen library preparation and sequencing

For RB-TnSeq experiments, gDNA was isolated from mutant library samples using a DNeasy Blood and Tissue kit (Qiagen, The Netherlands). PCRs were performed in 50 μl reactions with 20 μmol of each primer and 1 μl of template gDNA. All samples were amplified with a common reverse primer (oxp4027) and one of 16 barcoded forward primers (oxp4021-26 and oxp4156-4165). Cycling conditions were 98°C 4 min (×1); 98°C 30 s, 68°C 30 s, 72°C 30 s (×30); 72°C 5 min (×1). For each sample, five independent PCR amplifications were conducted, and equal volumes were pooled prior to purification with a Monarch PCR&DNA Cleanup Kit (NEB). Libraries were diluted to 75 pM and sequenced on an Ion Proton system following template preparation using the Ion Chef and Ion PI chip kit V3 (Thermo Scientific, Waltham, MA).

### Calculation of gene fitness values

Gene fitness values were calculated as previously described [25]; the code is available at https://bitbucket.org/berkeleylab/feba/. Briefly, a Perl script (MultiCodes.pl) is used to identify the barcode in each read and make a table containing all unique barcodes and how often each were seen. Next, a second Perl script (combineBarSeq.pl) takes the table of barcodes and combines it with the table of mapped mutants to make a new table of how often each mutant was seen. Lastly, an R script (BarSeqR.pl) combines this table with a genes table and uses a second custom R script (FEBA.R) to first calculate mutant fitness values and then gene fitness values followed by a measure of their reliability (*t*-score). For each experiment, strain fitness values are calculated as the log_2_ ratio of barcode relative abundance following library growth in each condition divided by the relative barcode abundance in the initial inoculum at time zero. The fitness of the gene is then calculated as the weighted average of the fitness values calculated for the mutants that have insertions within that gene. The data are normalized across the genome and independently for each scaffold so that the typical neutral gene has a fitness of zero meaning there is no difference in the number of barcode reads associated with that gene before and after treatment [25].

### Characterization of bacterial mutants

Mutants were generated using the pK18mobSacB vector for stable double recombinants or the pK19mob vector for integration mutagenesis and confirmed by Sanger sequencing [29]. All mutants were fluorescently labelled with Tn7 sfGFP and a total of 10^5^ cfu were co-inoculated onto pea plants in a 1:1 ratio with WT Rlv3841 fluorescently marked with Tn7 mCherry [30]. After 7 days, bacteria were retrieved from the rhizosphere and root (vortexed only) as previously described. An Amnis Cellstream (Luminex, Austin, TX) flow cytometer with autosampler, equipped with 488 nm, and 561 nm to excite sfGFP and mCherry respectively, was used to quantify each population (see SI Appendix).

## Results and discussion

### Development of RB-TnSeq in *R. leguminosarum*

To identify genes involved in rhizosphere persistence and root colonization, we generated a barcoded mariner transposon library in Rlv3841. Briefly, the *mariner* transposon delivery vector, pSAM_Rl, was converted to an RB-TnSeq vector by cloning random 20-nucleotide barcodes into the unique XhoI restriction site located within the transposon [31]. Barcoded transposons were introduced into Rlv3841 and INSeq used to identify the genomic locations of each insertion event and its associated barcode.

**Figure 1:**
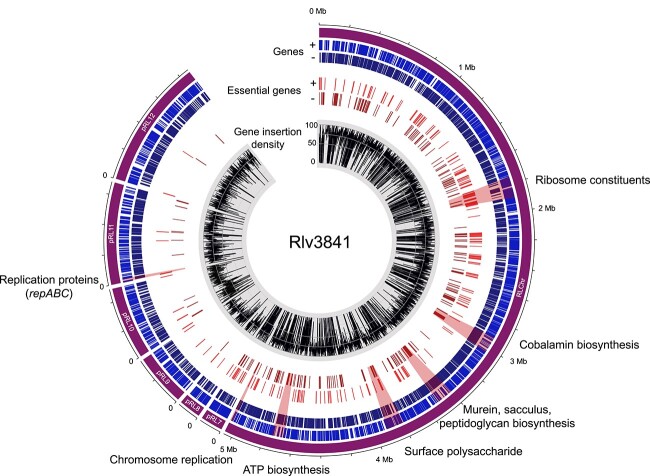
Genomic distribution of insertion mutants and essential genes in Rlv3841; outer to inner track: genomic position; gene distribution (each strand); essential genes (each strand); transposon insertion density per gene.

We identified 341 471 uniquely barcoded transposon insertions within the central 10% to 90% coding region of genes (Fig. 1). These mutants cover 82% of the potential 111 907 mariner insertion sites and are distributed across 6430 (90%) of protein-coding genes (Table S1). Only 720 genes lacked insertions, most of which are essential for Rlv3841 growth even in rich TY media (Table S2) [7]. Thus, our Rlv3841 transposon library contains mutants for most non-essential genes present in the genome, making it suitable for use in the genome-wide identification of genetic determinants contributing to bacterial fitness under various selective pressures.

### Requirements for bacterial rhizosphere fitness and root colonization

Rlv3841 insertion mutants were inoculated onto three host legumes (pea, lentil, and *Lathyrus*), a legume (alfalfa), and a non-legume (barley). Mutants were retrieved from the rhizosphere and root 7 days post inoculation (dpi). Input, rhizosphere, and root-colonized libraries were sequenced, and gene fitness values calculated as the log_2_ ratio of barcode abundance after growth in each condition divided by the barcode abundance in the initial inoculum at time zero [25]. Fitness values were normalized across the genome so that the typical gene has a neutral fitness value close to zero. Genes with a fitness value <−2 are considered to have severely disadvantaged phenotypes, with mutation resulting in a minimum 75% reduction in growth compared to the average mutant in the population. Values between −1 and −2 signify a moderate disadvantage (50%–75% growth reduction), whereas −0.42 to −1 represents a mild disadvantage (25%–50% growth reduction), and −0.42 to 0.42 a neutral phenotype. Fitness values above 0.42 suggest an advantageous phenotype meaning that these genes, when not mutated, hinder growth in that condition.

For input, rhizosphere, and root-colonized samples, an average of 322 000, 171 000, and 153 000 unique barcodes respectively were sequenced (Table S3). This enabled us to assign gene fitness values to 98% of genes for which we had mapped unique transposon insertion mutants (Table S4). The remaining 2% of genes were not assigned fitness values due to insufficient reads at time zero, indicating that the associated mutants are unfit *in vitro* and therefore account for a small proportion of the mutant library.

### Common gene requirements for rhizosphere fitness and root colonization

**Figure 2:**
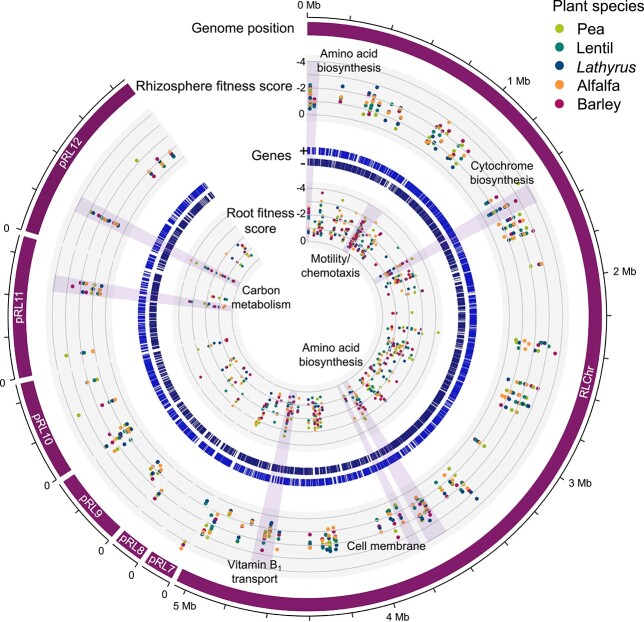
Genome-wide map of common rhizosphere persistence and root colonization genes; outer to inner track: genomic position; fitness value of genes commonly required for rhizosphere growth; gene distribution (each strand); fitness value of genes commonly required for root colonization.

Comparison of genes with a severe (fitness value < −2), moderate (−1 to −2), or mild (−0.42 to −1) disadvantage for growth in the rhizosphere or root colonization of all five plant species revealed 189 genes essential for optimal rhizosphere growth and 230 genes contributing to their root colonization (Fig. 2, Table S5). There is considerable overlap in the genetic requirements, with 111 genes common to both processes, suggesting that mutants affected in rhizosphere competitiveness are subsequently hindered in competitive root colonization. Furthermore, 77% of the rhizosphere specific genes and 66% of the root-colonization specific genes show a minimum 25% reduction in root colonization or rhizosphere growth, respectively, for at least three of the five plant species tested. We will therefore discuss the common requirements of rhizospheres fitness and root colonization collectively, highlighting rhizosphere or root-colonization specific determinants where appropriate.

#### Biosynthesis genes

The rhizosphere is widely considered a nutrient-rich environment capable of supporting microbial proliferation. This is primarily attributed to the presence of carbon-rich exudates released by plant roots [32]. Nonetheless, mutation of genes required for the biosynthesis of amino acids, vitamins (riboflavin and biotin), and ribonucleotides impairs bacterial growth in all five plant rhizospheres and leads to reduced root colonization (Table S6). Although these compounds are present in root exudates, their availability in the rhizosphere appears inadequate to sustain growth of auxotrophic mutants [33]. Variation in the severity of the growth impairment displayed by various amino acid auxotrophs also provides an indication of the most limiting amino acids for example, tryptophan, leucine, isoleucine, and valine auxotrophs showed the strongest phenotypes across all five plant rhizospheres with mutations in these pathways resulting in at least a 50% growth reduction. In addition to these metabolic compounds, de novo cytochrome *c* biogenesis (RL1436-7; *cycHJ* and RL1439-40; *cycLY*) contributes to rhizosphere fitness and root colonization.

#### Adaptation and regulation genes

Bacteria must navigate the heterogeneous environment that the rhizosphere provides, with variations in nutrient availability, pH, oxidative stress, and osmotic conditions at the microscale level [34, 35]. Mutation of several regulatory genes responsible for sensing and adapting to environmental stimuli were observed to hinder growth in the rhizosphere and colonization of all five plant species. These include the two-component regulatory systems FeuP/FeuQ and NtrB/NtrC, involved in iron and nitrogen homeostasis, respectively [36, 37]. Although plants were grown in nitrogen-free conditions to facilitate *Rhizobium*–legume signalling, this two-component system is likely to be important in soil environments where nitrogen availability is low.

*R. leguminosarum feuQ* mutants show reduced iron uptake [37], but the FeuPQ system is best characterized in *Sinorhizobium meliloti* where it also senses extracellular changes in osmolarity and, in response, alters transcription of 16 genes including *ndvA* [38]. NdvA transports cyclic ß-1-2-glucans to the cell surface where they play a key role in osmo-adaptation [39, 40]. In accordance with NdvA and NdvB, responsible for transport and biosynthesis of cyclic ß-1-2-glucan, respectively, were important for growth in the rhizosphere and root colonization. The rhizobial iron regulator, RirA, also contributed to root colonization [41]. Other regulators involved in rhizosphere adaptation include PhoU, a negative regulator of phosphate uptake and the alternative RNA polymerase sigma factor 54 RpoN. RpoN, required for *nif* gene expression and dicarboxylate transport during symbiosis, is also required for unknown processes in the rhizosphere [7].

#### Metabolism genes

Root exudates contain a variety of carbon sources that can effectively support bacterial growth. The ability of rhizobia to catabolize several of these provides a competitive advantage during nodulation of their host-legume [5, 8–10, 42, 43], though the precise stage of symbiosis at which this advantage arises remains unclear. We found that mutation of genes required for catabolism of erythritol (*eryB*; pRL120205, *eryR*; pRL120208, pRL120209; *tpiA2* and pRL120210; *rpiB2*), rhamnose (*rhaD*; pRL110415), and glycerol (*glyD*; pRL90074) hinders Rlv3841 proliferation in all five plant rhizospheres and subsequent root colonization. Additionally, catabolism of arabinose (*araD*; RL3614) promotes root colonization.

Transcriptome analyses of Rlv3841 during growth in plant rhizospheres demonstrate clear induction of genes involved in the Embden–Meyerhof–Parnas pathway [6]. Accordingly, mutation of RL0504 (*pgi*) RL4011 (*pgk*), RL0179 (*gpmA*) RL2239 (*eno*), and RL4605 (*galM*), encoding enzymes involved in this pathway, resulted in reduced rhizosphere fitness and root colonization. The transketolase CbbT (RL4006) also contributes to rhizosphere fitness and root colonization. Transketolases function in the pentose phosphate pathway, which maintains central carbon homeostasis, producing ribose 5-phosphate and erythrose 4-phosphate, precursors of nucleotides and histidine, and aromatic amino acids, respectively [44]. Although the Rlv3841 genome encodes four transketolases (RL2718, RL2719, RL4006 (*cbbT*), and pRL100453), only *cbbT* is commonly required, indicating it is the primary transketolase. Additionally, two other pentose phosphate pathway enzymes, RL4203 (*talB*) and RL2698 (*rpiA*), were important for colonization of all five plant species.

#### Transport genes

Considering that the Rlv3841 genome contains 269 genes involved in active uptake of solutes from the environment, very few are commonly required for growth in plant rhizospheres or root colonization. Exceptions to this are an ABC transporter of unknown class encoded by RL1003-04, a solute binding protein and associated ATP-binding component of an ABC transporter encoded by RL2659 and RL2660 respectively, and a nitrate/nitrite/cyanate ABC transporter encoded by RL4400-01, along with the associated solute binding protein encoded by RL4402. The latter is predicted to import the thiamine (vitamin B_1_) precursor hydroxymethylpyrimidine [45, 46]. Thiamine is an essential cofactor required for metabolism of branched-chain amino acids and carbohydrates [47]. Unlike many other bacteria, Rlv3841 does not have a pathway that allows de novo synthesis of thiamine and instead relies on a salvage pathway for which hydroxymethylpyrimidine is an intermediate [47]. Accordingly, two enzymes involved in the thiamine salvage pathway encoded by pRL110441 (*thiD*) and pRL110443 (*thiM*) were also required for rhizosphere growth and root colonization, respectively [47]. A copper efflux transporter, CopB (RL2435), was also important for colonization. Copper resistance mechanisms are widespread in plant-associated microbes and mutation of these have been shown to impair nodule symbioses [48, 49]. Transcriptomics analysis have also revealed up to an 8-fold induction of Rlv3841 *copB* during growth in plant rhizospheres and a 44-fold induction during growth in laboratory culture supplemented with pea root exudates [6]. Collectively, this suggests that plant root exudates contain a soluble form of copper that is perceived by rhizobia in the rhizosphere, with inability to initiate copper resistance mechanisms resulting in subsequent impairment to colonize plant roots and infect root nodules.

#### Cell surface genes

Properties of the bacterial cell surface influence adhesion, root colonization, and host-specificity during the establishment of *Rhizobium*–legume symbioses, while factors influencing cell wall integrity may alter survival in the rhizosphere [50, 51]. Lipopolysaccharides (LPSs) form a significant constituent of the Gram-negative outer membrane. Six enzymes of a LPS biosynthesis cluster, encoded by RL0813, RL0815, RL0818, RL0822, *gmd* (RL0825), and *fcl* (RL0826), were essential for colonization across plant species. Other LPS biosynthesis enzymes important for root colonization include a glycosyl transferase (RL1470), an LPS assembly protein (RL1567; *lptD*), a CMP KDO transferase (RL3439; *lpcB*) involved in the biosynthesis of the core region of LPS, and three enzymes involved in O-antigen biosynthesis encoded by pRL90053, pRL110056, and RL3667; the latter of which may also have a role in capsular polysaccharide biosynthesis [52–55].

Exopolysaccharides (EPSs), a major component of the cell surface that promotes cellular aggregation, also play a role in rhizosphere persistence and root colonization with the requirement of EPS biosynthesis genes *pssD* (RL3654), *pssO* (RL3663), and *lspL* (RL3677) [56, 57]. Other factors affecting the cell surface important for growth in the rhizosphere and root colonization include the outer membrane porin RopB (RL1589), a von Willebrand factor type A (pRL100386), and a filamentous hemagglutinin adherence factor precursor (RL4382). Rlv3841 RopB shares 98.6% identity to *Rlv* RCAM 1026 RopB, which form amyloid fibrils predicted to play a role in plant colonization [58]. This is consistent with the finding that bacterial amyloid fibrils in various bacteria, including CsgA in *E. coli*, form extracellular fimbriae called curli with direct roles as adhesins and biofilm constituents [59, 60]. Two genes, *dacF* (RL2477) and *dacC* (RL4363), involved in peptidoglycan biosynthesis promote root colonization.

#### Chemotaxis and motility genes

Plant photosynthates secreted into the rhizosphere form chemical gradients that are perceived by bacteria, which respond by moving along these gradients towards roots. Requirement for chemotaxis and flagella-mediated motility during bacterial root colonization is well established in the literature for various soil bacteria [11, 12, 61, 62]. The Rlv3841 genome contains 92 genes predicted to have a role in chemotaxis and flagella-mediated motility and mutation of 24 of these genes results in reduced root colonization of all five plant species (Table S7). These include genes belonging to the *che1* chemotaxis cluster, the flagella biosynthesis cluster, a methyl-accepting chemotaxis receptor *mcpE* (RL0564), and transcriptional regulators *visN* (RL0696), *rem* (RL0727), and *flbT* (RL0732). Although Rlv3841 contains two chemotaxis gene clusters (*che1* and *che2*), the *che1* cluster is the major pathway controlling chemotaxis with mutants impaired in competitive nodulation of pea [11].

#### Plant-specific genes

**Figure 3:**
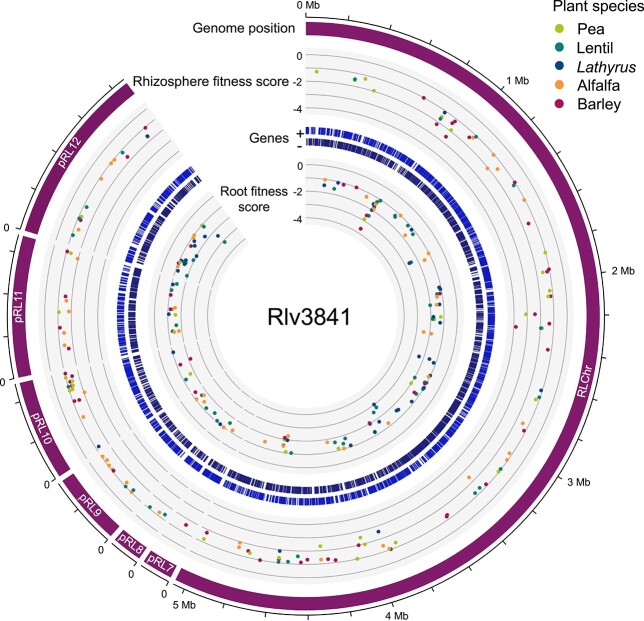
Genome-wide map of plant-specific rhizosphere persistence and root colonization genes; outer to inner track: genomic position; fitness value of genes commonly required for rhizosphere growth; gene distribution (each strand); fitness value of genes commonly required for root colonization.

**Figure 4:**
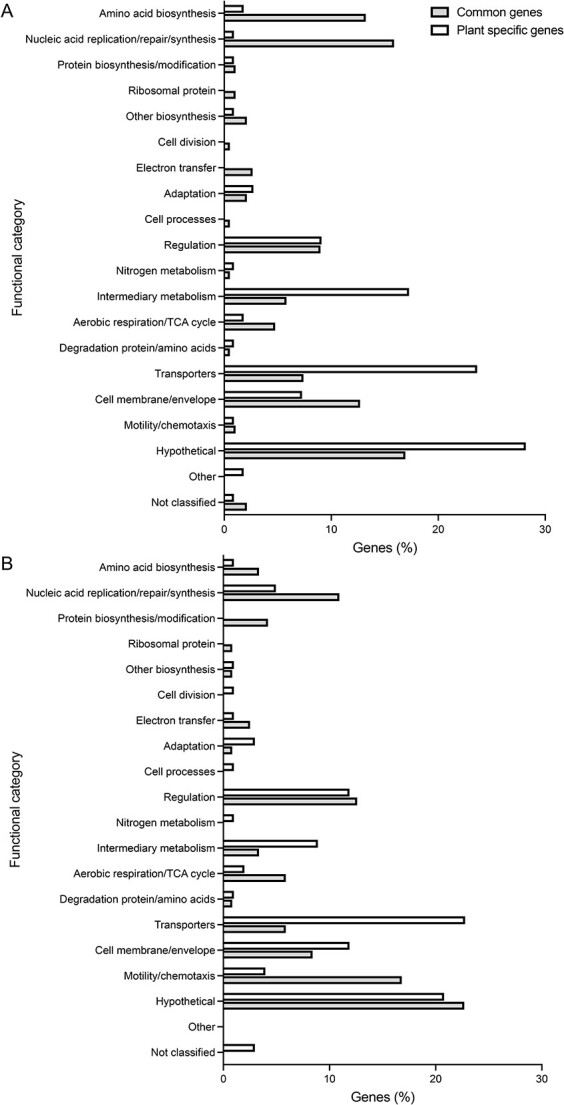
Functional classification of common and plant-specific genes; (A) rhizosphere growth; (B) root colonization; functional classifications are based on Riley codes [69]; genes are listed in Tables S8 and S9.

Comparison among datasets enabled us to identify genes that may have plant-specific roles in rhizosphere fitness or root colonization. Genes with a fitness value <−1 for either one or a subset of the plant species tested, but a fitness value greater than −0.42 for all other plant species were identified. Given that we pooled all samples for each plant species and therefore lack independent replicates, these genes were then further filtered based on their associated *t*-score, a measure of consistency in the strain fitness values used to calculate the gene fitness value, to retain those which have high confidence in the associated fitness value. We identified 110 genes to have plant-specific phenotypes for growth in the plant rhizospheres tested, nine of which also affected subsequent root colonization (rhizosphere progressive) (Fig. 3, Table S8). These genes predominately had unknown functions (28%), roles in transport (24%), intermediary metabolism (17%), and regulation (9%) in contrast to the 189 genes commonly required for rhizosphere growth where, following hypothetical proteins (17%), genes predominantly had roles in nucleic acid replication/repair/synthesis (16%), amino acid biosynthesis (13%), and cell membrane/envelope (13%) (Fig. 4A). Root exudate composition varies among plant species, and it is therefore not surprising that numerous plant-specific genes required for rhizosphere fitness are implicated in nutrient uptake from the rhizosphere.

For colonization, a further 101 genes display plant-specific phenotypes (Fig. 3, Table S9). Those with known functions primarily had roles in transport (23%), cell membrane/envelope modification (12%), protein biosynthesis/modification (12%), and intermediary metabolism (9%). In contrast, common determinants of root colonization, where motility/chemotaxis (17%), regulation (13%), nucleic acid replication/repair/synthesis (11%), and cell membrane/envelope (8%) accounted for the largest functional categories after hypothetical genes. Motility and chemotaxis are prerequisites for root colonization and therefore, expectedly, they accounted for 17% of genes commonly required for colonization for all five plant species (Fig. 4B). Only two genes with putative roles in these processes showed plant-specific phenotypes. These were RL0949 encoding a methyl-accepting chemotaxis protein McpJ important for lentil and pRL120064 encoding the flagellar hook protein FlgE2 important for *Lathyrus* colonization. The identification of McpJ as uniquely required for lentil colonization suggests that it recognizes an amino acid or small carbohydrate present in lentil root exudates, but not pea, *Lathyrus*, alfalfa or barley [63]. The Rlv3841 genome encodes two FlgE proteins, FlgE1 (RL0728) and FlgE2 (pRL120064), the former of which was essential for colonization of all five plant roots. The role of FlgE2 specifically for colonization of *Lathyrus* is unclear but it might play a direct role in attachment to the root surface of *Lathyrus*.

Genes involved in modification of the cell surface also account for a substantial proportion of plant-specific genes implicated in root colonization. These genes could alter the adhesive properties of the cell with different requirements in polysaccharide structure or cell surface proteins possibly reflecting variation in root surface properties between plant species. A glycosyl transferase (pRL90136), outer membrane protein encoded by pRL110591, a MipA family outer membrane protein encoded by RL0955, and an OmpA family outer membrane protein encoded by RL2752 were specifically required for colonization of lentil. For barley root colonization, a transmembrane attachment related protein encoded by pRL90312 was important.

We did not find any genes uniquely required for growth in the rhizosphere or colonization of host legumes (pea, lentil, and *Lathyrus*) compared to non-hosts (alfalfa and barley). In addition, several genes previously implicated in Rlv3841 attachment to pea root hairs showed neutral phenotypes for pea-root colonization. These include *gmsA* (RL1661), *celA* (RL1646), and *pssA* (RL3752), suggesting the existence of alternative mechanisms for rhizobial attachment to root hairs (1–2 h postinoculation) and long-term colonization over the entire root surface (7 dpi) [64].

It is entirely plausible that genes with milder fitness contributions, for example those with a fitness value of −0.42 representing a 25% reduction in growth, have biological significance for plant-specific phenotypes. However, previous RB-TnSeq screens have shown that there is higher statistical confidence in fitness estimates for genes with stronger fitness contributions [65]. Conducting multiple replicates under a given condition to see if genes with weaker contributions are consistently identified would provide greater support in identification of these genes with milder plant-specific phenotypes.

### RB-TnSeq facilitates finer resolution of mutant phenotypes during initial stages of symbiosis

**Figure 5:**
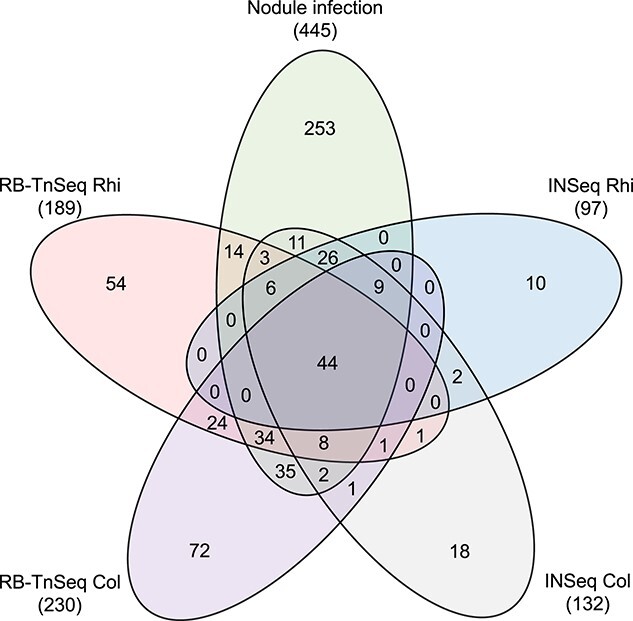
Venn diagram showing comparison of genes identified in RB-TnSeq and INSeq screens as contributing to Rlv3841 rhizosphere (rhi) growth, root colonization (col), or pea-nodule infection; genes are listed in Table S10.

Our previous INSeq screen, that assessed the fitness contribution of Rlv3841 genes during multiple stages of symbiosis with pea, found 542 genes critical for nodule infection [7]. We calculated gene fitness values for 445 of these, 67 lacked mapped insertion mutants, while 30 had insufficient reads at time zero suggesting that these mutants are debilitated in the input library (Table S10). Comparison of these 445 genes to those identified in this study as commonly required for Rlv3841 growth in plant rhizospheres or root colonization revealed 155 genes in common, 83 of which were previously classified as neutral for pea rhizosphere growth and root colonization (Fig. 5) [7]. In fact, our RB-TnSeq screens reveal that mutations in 247 (56%) of these 445 genes result in at least a 50% reduction in Rlv3841 growth in the pea rhizosphere or root colonization (Table S10). Collectively these results suggest that mutations in these genes initially affect Rlv3841 competitiveness during growth in the rhizosphere or root colonization, and that this impairment persists throughout symbiosis development.

To ascertain gene essentiality in our initial INSeq screen, we used a Hidden Markov Model, designating genes important for growth if they have <1% of the mean read count [66]. In contrast, analysis of RB-TnSeq data involves comparison of gene read number before and after treatment, thereby facilitating the identification of genes with milder, yet still biologically significant, contributions. One example is the identification of 24 genes involved in chemotaxis and flagella-mediated motility as common determinants of root colonization, all of which were determined to have no effect on root colonization in our INSeq screen [7]. Other examples of processes identified in this study to initially be important for Rlv3841 growth in plant rhizospheres and not at later stages of symbiosis include purine ribonucleotide and cytochrome *c* biosynthesis, arabinose and rhamnose catabolism, and various surface polysaccharides modifications (Table S10). Though not incorrect, we believe that the Hidden Markov Model can be overly conservative, assigning neutral classifications to genes that have biologically significant effects. This makes analysis methods in which the frequency of mutants is compared before and after growth preferable for assessing mutant fitness when the selective pressure in which they are grown limits the generations of growth through which they can undergo.

Transposon screening approaches, including RB-TnSeq and INSeq, are limited as methods by the fact that they are unlikely to identify factors that can be complemented *in trans*. An example of which is a strain impaired in siderophore production, widely known to be important during competition in the rhizosphere [67], which would not be identified in RB-TnSeq experiments because near isogenic strains present in the mutant population are still able to synthesize and export siderophores. This would also apply to genes involved in the production of quorum sensing signals, antimicrobial compounds, or metabolic intermediates that may be taken up and utilized by a nonproducing strain.

### Validation of RB-TnSeq predictions

**Figure 6:**
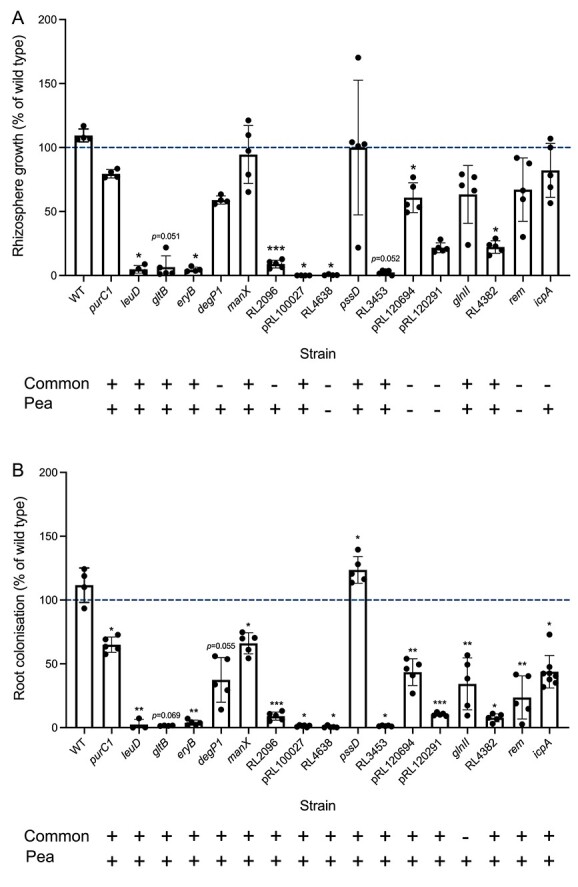
Validation of experimental phenotypes predicted from RB-TnSeq screens; competition for (A); rhizosphere growth and (B); root colonization of mutants with wild type from 1:1 co-inoculation (total, 10^5^ cfu) of pea plants retrieved at 7 dpi (*n* ≥ 3); RB-TnSeq phenotype for either all plants or pea is indicated by “+” if required and “–” if not; statistical significance was assessed by paired *t*-test; ^*^P < .05; ^**^P < .01; ^***^P < .001; error bars show ± SD.

Seventeen genes identified by RB-TnSeq to be important for growth in the rhizosphere or colonization of all five plant species were mutated and their mutant phenotypes assessed in co-inoculation plant assay with wild type Rlv3841 on pea (Fig. 6, SI Appendix, Tables 5 and 6). For growth in the rhizosphere, 10 genes show the expected phenotype, with at least a 20% reduction in growth relative to wild type (Fig. 6A). Mutants in *icpA* also show the expected trend, with an average 18% decrease in rhizosphere growth. Conversely, *manX* and *pssD* mutants demonstrated comparable growth to wild type despite having gene fitness values below −1.7 for growth in all five plant rhizospheres. Although *rem* gene fitness values remained neutral (above −0.42) across all five plant rhizospheres, *rem* mutants exhibited an average 33% decrease for growth in the pea rhizosphere, though this was not deemed statistically significant. Mutation of RL4638, pRL120291, and pRL120694 significantly impairs growth, even though these genes are not considered core determinants of rhizosphere fitness. Rhizosphere gene fitness values for these genes varied across plant species: RL4638 ranged from −0.23 to −0.95, pRL120694 from 0.27 to −0.5, and for pRL120291, values were <−1.5 for growth in three plant rhizospheres (*Lathyrus*, alfalfa, and barley), but greater than −0.42 in the remaining (pea and lentil). The clear impairment of pRL120291 mutants for growth in the pea rhizosphere suggests that pRL120291 may in fact be a core determinant of rhizosphere fitness. For root colonization, all mutants showed a clear impairment apart from *pssD*, which showed a significant increase in colonization (Fig. 6B).

## Conclusion

**Figure 7:**
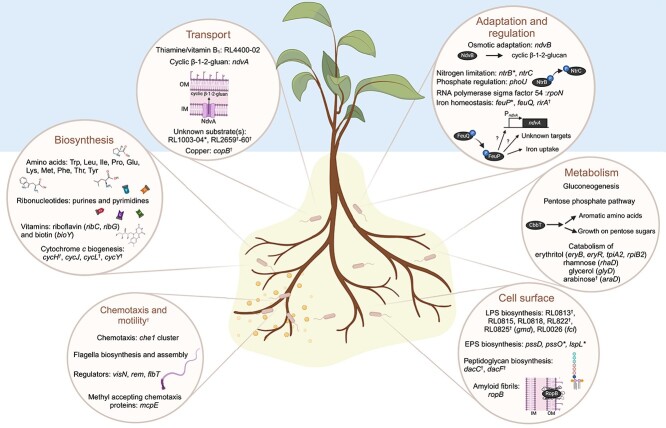
Summary of Rlv3841 common determinants for growth in plant rhizospheres and root colonization; genes have a rhizosphere progressive phenotype for all plant species unless highlighted with a “^*^” to demonstrate a rhizosphere specific phenotype or a “^†^” to denote a colonization specific phenotype; full gene list is available in Table S5; created with BioRender.com.

To establish an effective symbiosis, rhizobia must first survive in soil and compete with other microbiome members to colonize their host legume. It is predicted that a single rhizobial cell in soil has just a one in a million chance of finding its symbiotic host, and yet few studies have focused on identifying genes involved in these initial processes that precede nodule infection itself [68]. Here we reveal a complex network of genetic determinants fundamental for rhizobial persistence in diverse plant rhizospheres and subsequent root colonization. Common processes are summarized (Fig. 7), highlighting a need to synthesize essential compounds, adapt metabolic function, respond to external stimuli, withstand various stresses, and for root colonization, chemotactic motility. Numerous genes previously associated with later stages of symbiosis, such as nodule infection or bacteroid development itself, were found to play crucial roles in these initial phases, emphasizing both their importance and the sensitivity of the RB-TnSeq pipeline. Furthermore, this study sheds light on the plant-specific factors affecting bacterial growth in plant rhizospheres and root colonization. The increased requirement for genes involved in transport and intermediary metabolism likely reflects variation in resource availability among the plant rhizospheres due to altered root exudate composition, while an increased demand of cell membrane modification genes may reflect variation of root surface properties. These insights provide a greater understanding of how bacterial associations develop across diverse plant species. Identifying core genes responsible for rhizosphere adaption and root colonization will aid future studies in developing a minimal synthetic genome for a plant-colonizing bacterium, while also providing engineering targets for the development of strains with enhanced rhizosphere competitiveness and root colonizing abilities. Such advancements will facilitate the effective exploitation of plant-beneficial bacteria for agricultural gain.

## Supplementary Material

Supplementary_data_tables_wrae072

Supplementary_appendix_revised_wrae072

## Data Availability

The data supporting the findings of this study are available in this article, Supplementary Data Tables, and its SI Appendix. Raw sequencing for indexing of the mutant library and RB-TnSeq screens is available under Bio Projects PRJNA1052635 and PRJNA1053904, respectively.
