# Supplementary material for: Rhizobium determinants of rhizosphere persistence and root colonization
Source: ISME J. 2024 May 1;18(1):wrae072. doi: 10.1093/ismejo/wrae072 (PMC11103875; doi:10.1093/ismejo/wrae072)
Supplement: Supplementary_appendix_revised_wrae072 [file supplementary_appendix_revised_wrae072.docx]

**Supporting Information Appendix**

***Rhizobium* determinants of rhizosphere persistence and root colonisation**

Hayley E. Knights^1^, Vinoy K. Ramachandran, Beatriz Jorrin, Raphael Ledermann, Jack D. Parsons, Samuel T.N. Aroney and Philip S. Poole

Department of Biology, University of Oxford, OX1 3RB, Oxford, United Kingdom

^1^To whom correspondence may be addressed. Email: [hayley.knights@biology.ox.ac.uk](mailto:hayley.knights@biology.ox.ac.uk).

**Supplementary Data Tables**

Supplementary data tables can be found in the excel file named ‘Supplementary Data Tables’.

**Table S1.** 6,430 protein coding genes containing reliably mapped insertions within the central 10-90% region.

**Table S2.** 720 unhit protein coding genes lacking insertion mutants in the Rlv3841 barcoded transposon pool.

**Table S3.** Number of unique barcoded mutants retrieved for each sample.

**Table S4.** Gene fitness values for 6,313 Rlv3841 genes during growth in the rhizosphere and colonisation of pea, lentil, *Lathyrus*, alfalfa and barley.

**Table S5.** 189 genes commonly required for growth in plant rhizospheres (111 rhizosphere progressive) and 119 genes necessary for root colonisation.

**Table S6.** RB-TnSeq identified metabolic phenotypes in Rlv3841 during growth in the rhizosphere and colonisation of pea, lentil, *Lathyrus*, alfalfa and barley.

**Table S7.** Phenotypic summary of 92 Rlv3841 chemotaxis and flagella mediated motility genes.

**Table S8**. Genes with plant specific phenotypes for rhizosphere growth. Gene must have FV <-1 and t-score <-2.5 or FV <-2 and t-score <-3 and FV >-0.42 for rhizosphere growth and root colonisation of all other plan species.

**Table S9.** Genes with plant specific phenotypes for root colonisation. Gene must have FV <-1 and t-score <-2.5 or FV <-2 and t-score <-3, and FV >-0.42 for rhizosphere growth and root colonisation of all other plant species.

**Table S10.** Comparison of 542 protein coding genes identified by INSeq to contribute to Rlv3841 competitive nodulation of pea.

**SI Appendix Tables**

**SI Appendix, Table 1.** Bacterial strains used in this study.

| **Species** | **Strain** | **Description** | **Source** |
| --- | --- | --- | --- |
| *Escherichia coli* | DH5α | Competent *E. coli* strain carrying the following mutations; F*- deoR endA1 recA1 relA1 gyrA96 hsdR17(rk-mk+) supE44 thi-1 - phoA Δ(lacZYA-argF)*U169 Φ80*lacZ*ΔM15 λ | Bioline |
|  | TransforMax^TM^ EC100D^TM^ *pir*-116 | Electrocompetent *E. coli* strain carrying the following mutation; F- *mcrA* ∆(*mrr*-hsdRMS-*mcr*BC) ϕ80d*lac*Z∆M15 ∆*lac*X74 *rec*A1 *end*A1 *ara*D139 ∆(*ara*, *leu*)7697 *gal*U *gal*K λ– *rps*L *nup*G *pir-*116(DHFR). | Lucigen |
| *Rhizobium leguminosarum* | Rlv3841 | *Rhizobium leguminosarum* biovar *viciae* 3841; Str^R^ derivative of strain 300. | (1) |
|  | LMB143 | Tn5 insertion in RL4085 (*gltB*), transduced into Rlv3841. | (2) |
|  | RU4164 | Tn5 insertion in RL2096, transduced into Rlv3841. | This work |
|  | RU4241 | Rlv3841 pK19 mutant of RL4705 (*leuD*) made with pRU2135. | This work |
|  | RU4286 | Rlv3841 pK19 mutant of RL0033 (*manX*) made with pRU2155. | This work |
|  | RU4300 | Rlv3841 pK19 mutant of RL1440 (*degP1*) made with pRU2165. | This work |
|  | RU4360 | Rlv3841 pK19 mutant of pRL100162 (*nifH*). | (3) |
|  | OPS1603 | Rlv3841 pK19 mutant of pRL120205 (*eryB*). | (4) |
|  | OPS1607 | Rlv3841 pK19 mutant of RL2606 (*purC1*). | (4) |
|  | OPS1608 | Rlv3841 pK19 mutant of RL3549 (*glnII*). | (4) |
|  | OPS1609 | Rlv3841 pK19 mutant of RL3654 (*pssD*). | (4) |
|  | OPS1782 | Rlv3841 pK19 mutant of RL4382. | (5) |
|  | OPS1907 | Rlv3841 pK19 mutant of RL3453. | (5) |
|  | OPS1972 | Rlv3841 pK19 mutant of pRL120694 made with pOPS1282. | This work |
|  | OPS1977 | Rlv3841 pK19 mutant pRL120291 made with pOPS1285. | This work |
|  | OPS1978 | Rlv3841 pK19 mutant of (*rem*) made with pOPS1287. | This work |
|  | OPS2043 | Rlv3841 markerless in-frame deletion of pRL100027 made with pOPS1337. | This work |
|  | OPS2047 | Rlv3841 markerless in-frame deletion of RL4638 made with pOPS1343. | This work |
|  | OPS2108 | Rlv3841 markerless in-frame deletion of RL0685 (*icpA*) made with pOPS1411. | This work |
|  | OPS2452 | Rlv3841 integration with plasmid pOPS1526. | (6) |
|  | OPS2453 | Rlv3841 integration with plasmid pOPS1531. | (6) |
|  | OPS3662 | OPS1978 (Rlv3841 ∆RL0727; *rem*) integration with plasmid pOPS1526. | This work |
|  | OPS3663 | OPS1977 (Rlv3841 ∆pRL120291) integration with plasmid pOPS1526. | This work |
|  | OPS3664 | OPS1609 (Rlv3841 ∆RL3654; *pssD*) integration with plasmid pOPS1526. | This work |
|  | OPS3665 | OPS1608 (Rlv3841 ∆RL3549; *glnII*) integration with plasmid pOPS1526. | This work |
|  | OPS3666 | RU4300 (Rlv3841 ∆RL1440; *degP1*) integration with plasmid pOPS1526. | This work |
|  | OPS3667 | RU4286 (Rlv3841 ∆RL0033) integration with plasmid pOPS1526. | This work |
|  | OPS3668 | OPS1972 (Rlv3841 ∆pRL120694) integration with plasmid pOPS1526. | This work |
|  | OPS3669 | OPS1907 (Rlv3841 ∆RL3453) integration with plasmid pOPS1526. | This work |
|  | OPS3670 | RU4241 (Rlv3841 ∆RL4705; *leuD*) integration with plasmid pOPS1526. | This work |
|  | OPS3672 | RU4164 (Rlv3841 ∆RL2096) integration with plasmid pOPS1526. | This work |
|  | OPS3673 | LMB143 (Rlv3841 ∆RL4085; *gltB*) integration with plasmid pOPS1526. | This work |
|  | OPS3675 | RU4360 (Rlv3841 ∆pRL100162; *nifH*) integration with plasmid pOPS1531. | This work |
|  | OPS3676 | OPS1782 (Rlv3841 ∆RL4382) integration with plasmid pOPS1526. | This work |
|  | OPS3677 | OPS1607 (Rlv3841 ∆RL2606; *purC1*) integration with plasmid pOPS1526. | This work |
|  | OPS3678 | OPS1603 (Rlv3841 ∆pRL120205; *eryB*) integration with plasmid pOPS1526. | This work |
|  | OPS3698 | OPS2043 (Rlv3841 ∆pRL100027) integration with plasmid pOPS1526. | This work |
|  | OPS3700 | OPS2047 (Rlv3841 ∆RL4638) integration with plasmid pOPS1526. | This work |
|  | OPS3701 | OPS2108 (Rlv3841 ∆RL0685; *icpA*) integration with plasmid pOPS1526. | This work |

**SI Appendix, Table 2.** Plasmids used in this study.

| **Plasmid name** | **Description** | **Source** |
| --- | --- | --- |
| pSAM_RI | pSAM_Rl *mariner* transposon vector in *E. coli* SM10λ*pir* background; Kan^r^ /Neo^r^, Amp^r^ | (7) |
| pSAM_RI_RBP1 | *E. coli* pool collectively carrying millions of the modified pSAM_RI vectors containing a randomly barcoded transposon. | This work |
| pRK2013 | Helper plasmid for triparental conjugation; Kan/Neo^r^ | (8) |
| pK18mobSacB | pK18 based mobilizable plasmid for double recombination in Rhizobium, suicide vector on sucrose; Kan/Neo^R^. | (9) |
| pK19mob | Mobilisable vector used for integration mutagenesis in Rhizobium; Kan/Neo^R^ | (9) |
| pRU2135 | PCR amplified internal fragment of Rlv3841 RL4705 (*leuD*) (with p1588 and p1589) BD cloned into SpeI digested pK19mob). | This work |
| pRU2155 | PCR amplified internal fragment of Rlv3841 RL0033 (*manX*) (with p1518 and p1519) BD cloned into pK19mob. | This work |
| pRU2165 | PCR amplified internal fragment of Rlv3841 RL1440 (*degP1*) (with p1539 and p1540) BD cloned into pK19mob. | This work |
| pOPS1282 | PCR amplified internal fragment of Rlv3841 pRL120694 (with oxp3320-oxp3321) BD cloned into HindIII digested pK19mob. | This work |
| pOPS1285 | PCR amplified internal fragment of Rlv3841 pRL120291 (with oxp3317-oxp3327) BD cloned into HindIII digested pK19mob. | This work |
| pOPS1287 | PCR amplified internal fragment of Rlv3841 RL0727 (with oxp3322-oxp3323) BD cloned into HindIII digested pK19mob. | This work |
| pOPS1337 | PCR amplified up- (oxp3496/oxp3497) and down-stream (oxp3494/oxp3495) flanking regions of RL4638 HiFi cloned into HindIII and BamHI digested pK18mobsacB. | This work |
| pOPS1343 | PCR amplified up- (oxp3512/oxp3513) and down-stream (oxp3510/oxp3511) flanking regions of RL4638 HiFi cloned into HindIII and BamHI digested pK18mobsacB. | This work |
| pOPS1526 | Tn7 sfGFP plasmid: J23104-RBstd-sfGFP-DT16-pOGG276 (pUC18R6K-miniTn7T-Gm). | (6) |
| pOPS1531 | Tn7 mCherry plasmid: J23104-RBStd-mCherryI-DT16-pOGG276 (pUC18R6K-miniTn7T-Gm). | (6) |
| pOPS1411 | PCR amplified up- (oxp3769/oxp3770) and down-stream (oxp3771/oxp3772) flanking regions of RL0685 (*icpA*) HiFi cloned into SmaI digested pK18mobsacB. | This work |

BD = BD In-Fusion^TM^ PCR Cloning Kit (Takara Bio)

HiFi = NEBuilder^®^ HiFi DNA Assembly Cloning Kit (New England Biolabs)

**SI Appendix, Table 3.** TY+ media recipe for 1 L. Based on a combination of TY and Universal minimal salts media (10).

| **Compound** | **Amount** | **Final concentration in TY +** |
| --- | --- | --- |
| Tryptone | 2.5 g | 35.2 mM |
| Yeast extract | 1.5 g | 5.5 mM |
| 1M K_2_HPO_4_ | 0.25 mL | 0.25 mM |
| MgSO_4._7H_2_O | 0.25 g | 1 mM |
| NaCl | - 1. g | 1.7 mM |
| MOPS | 2.01 g | 10 mM |
| Trace elements | 0.5 mL | - |
| Calcium stock | 5.42 mL | - |
| Iron stock | 500 µL | - |
| Vitamin stock | 1 mL | - |
| 1M Glucose | 8 mL | 10 mM |
| 1M Succinate | 16 mL | 20 mM |
| 1M Ammonium | 8 mL | 10 mM |
| 1M Glutamate | 8 mL | 10 mM |
| **Trace elements – dissolved in 1L GDW** | | |
| EDTA-Na_2_ | 0.375 g | 0.5 µM |
| ZnSO_4_7H_2_O | 0.16 g | 0.3 µM |
| NaMoO_4_ | - 1. g | 0.4 µM |
| H_3_BO_3_ | 0.25 g | 2.0 µM |
| MnSO_4_4H_2_O | 0.2 g | 0.45 µM |
| CuSO_4._5H_2_O | 0.02 g | 0.04 µM |
| CoCl_2_6H_2_O | 1 g | 2.1 µM |
| **Calcium stock – dissolve in 100 mL** | | |
| CaCl_2._2H_2_O | 6.66 g | 3.25 mM |
| **Iron stock – dissolved in 100 mL of 1M HCL** | | |
| FeSO_4._7H_2_O | 1.2 g | 0.02 µM |
| **Vitamin stock – dissolved in 1L GDW** | | |
| Thiamine hydrochloride | 1 g | 3.0 µM |
| D-Pantothenic acid Ca salt | 2 g | 4.2 µM |
| Biotin | 100 mg | 0.4 µM |

**SI Appendix, Table 4.** Primers and oligos used in this work.

| **Name** | **Sequence 5’ 🡪 3’** | **Description** | **Reference** |
| --- | --- | --- | --- |
| Oligonucleotide pool | GATGTCCACGAGGTCTCTNNNNNNNNNNNNNNNNNNNNCGTACGCTGCAGGTCGAC | Oligonucleotide pool of random barcodes flanked by universal primer binding sites | This work |
| Ion Torrent BioSam | /BiotinTEG/CGGTTCGCTTGCTGTCCAT AAAACC | Ion Torrent BioSAM with 5' Biotin TEG | (7) |
| M12 Top | CTGTCCGTTCCGACTACCCTCCCGAC | M12 adaptor top | (11) |
| M12 Bottom | GTCGGGAGGGTAGTCGGAACGGACAG | M12 adaptor bottom | (11) |
| INSeq_Adpt_Top | AGATCGGAAGAGCGTCGTGTAGGGAA | INSeq adaptor top | (7) |
| INSeq_Adpt_Bottom | TTCCCTACACGACGCTCTTCCGATCTNN | INSeq adaptor bottom | (7) |
| IT_trP1_FP | CCTCTCTATGGGCAGTCGGTGATTTCCCT ACACGACGCTCTTCCGATCT | Ion Proton universal 3’ sequencing adaptor | (7) |
| IT_A_FP_1 | CCATCTCATCCCTGCGTGTCTCCGACTCA GCTAAGGTAACGATATAAAACCGCCC AGTCTACTCGAGGG | Ion Xpress 5’ barcoded sequencing adaptor | (7) |
| Tn5 | GAACGTTACCATGTTAGGAGGT | Primer for Tn5 mapping | (12) |
| pK19/18mob A | ATCAGATCTTGATCCCCTGC | Rev primer for PCR amplification for region of pK19/18mob | This work |
| pK19/18mob B | GCACGAGGGAGCTTCCAGGG | Fwd primer for PCR amplification for region of pK19/18mob | This work |
| oxp2058 | TTCGCTCGATTTTACCAAGC | Fwd primer to validate RL4382 pK19 mutant | (5) |
| oxp2059 | AGATTGCGGACCGACGT | Rev primer to validate RL4382 pK19 mutant | (5) |
| oxp2374 | CAAGGCGCTTGTCCAT | Fwd primer to validate RL3654 (*pssD*) pK19 mutant | (4) |
| oxp2375 | AGATTTCATGCCGATC | Fwd primer to validate RL3549 (*glnII*) pK19 mutant | (4) |
| oxp2376 | ATTCTCGAAGAACTCGACTG | Fwd primer to validate RL2606 (*purC1*) pK19 mutant | (4) |
| oxp2381 | TGCGTCACGGTTTCCCCGAC | Fwd primer to validate pRL120205 (*eryB*) pK19 mutant | (4) |
| oxp2702 | GCGGTTCGGCCTGGTTCAAT | Rev primer to validate RL2606 (*purC1*) pK19 mutant | (4) |
| oxp2703 | GCCAGTTCGCGGTTGGTGTT | Rev primer to validate RL3549 (*glnII*) pK19 mutant | (4) |
| oxp2711 | CGAAATGGTTGAGGCGCCCA | Rev primer to validate pRL120205 (*eryB*) pK19 mutant | (4) |
| oxp2713 | ACCTGGCCACGGCAATTCCT | Rev primer to validate RL3654 (*pssD*) pK19 mutant | (4) |
| oxp2864 | TAACTGACGTGAGCAGCGAC | Fwd primer to validate RL3453 pK19 mutant | (5) |
| oxp2865 | CGCCGACGACTATCTTTCCA | Rev primer to validate RL3453 pK19 mutant | (5) |
| oxp3317 | GCAGGCATGCAAGCTACGTCGCGGTTCAGAGC | Rev primer for amplification of pRL120291 internal fragment | This work |
| oxp3320 | TGATTACGCCAAGCTCTCGCACGCACTGAACG | Fwd primer for amplification of pRL120694 internal fragment | This work |
| oxp3321 | GCAGGCATGCAAGCTCGGACGGCTGAATTCGC | Rev primer for amplification of pRL120694 internal fragment | This work |
| oxp3322 | TGATTACGCCAAGCTTAGCTGCAGTCGAGGCC | Fwd primer for amplification of RL0727 internal fragment | This work |
| oxp3323 | GCAGGCATGCAAGCTGCGCTTGGAATCGACCG | Rev primer for amplification of RL0727 internal fragment | This work |
| oxp3327 | TGATTACGCCAAGCTGAGTTGGGCCTGACGTACC | Fwd primer for amplification of pRL120291 internal | This work |
| oxp3407 | GTCCCCGGTCTTCGTATGCCGTCTTCT GCTTGGCGCGCCCTCGAGGATGTCC ACGAGGTCTCT | Oligonucleotide pool amplification primer (forward) | This work |
| oxp3408 | ATTCCGGTTCGCTTGCTGTCCATAAAACC GCCCAGTCTACTCGAGGGGTCGACCTG CAGCGTACG | Oligonucleotide pool amplification primer (reverse) | This work |
| oxp3494 | CGAGCTCGGTACCCGGGTTCGATCTTTGGCCTGAC | Fwd primer for amplification of pRL100027 downstream flanking region for mutagenesis | This work |
| oxp3495 | GTACTAGTAAACCGCCGACATAATCG | Rev primer for amplification of pRL100027 downstream flanking region for mutagenesis | This work |
| oxp3496 | CGGCGGTTTACTAGTACCCTTGTGCATCAGCGAAG | Fwd primer for amplification of pRL100027 upstream flanking region for mutagenesis | This work |
| oxp3497 | AACGACGGCCAGTGCCAAATTGGCAACGCCTTGGC | Rev primer for amplification of pRL100027 upstream flanking region for mutagenesis | This work |
| oxp3510 | ATTCGAGCTCGGTACCCGGGTCTAATGCCGGGATGCGAAC | Fwd primer for amplification of RL4638 downstream flanking region for mutagenesis | This work |
| oxp3511 | GCCCATTTCCACTAGTGTCTACGCAGGCTGAAGG | Rev primer for amplification of RL4638 downstream flanking region for mutagenesis | This work |
| oxp3512 | AGACACTAGTGGAAATGGGCAAAACGGTCTC | Fwd primer for amplification of RL4638 upstream flanking region for mutagenesis | This work |
| oxp3513 | TAAAACGACGGCCAGTGCCAGCTGGCCGGAACAATGGC | Rev primer for amplification of RL4638 upstream flanking region for mutagenesis | This work |
| oxp3550 | TCCAGCCAGAACCGTCCGC | Fwd primer to validate pRL120291 pk19 mutant | This work |
| oxp3551 | TGCCGCGACCGTCAGACTG | Rev primer to validate pRL120291 pK19 mutant | This work |
| oxp3554 | TTTGGAGAAAGCCCGCGC | Fwd primer to validate pRL120694 pk19 mutant | This work |
| oxp3555 | GGCTCGTCATTGCGGAAGG | Rev primer to validate pRL120694 pK19 mutant | This work |
| oxp3556 | CGAGAATTTCCGCTCCGCC | Fwd primer to validate RL0727 pk19 mutant | This work |
| oxp3557 | GTCGTGGTGGCACTGTTGG | Rev primer to validate RL0727 pK19 mutant | This work |
| oxp3642 | TTGTGAGAGAGTGGGCCGC | Fwd primer to validate pRL100027 deletion mutant | This work |
| oxp3643 | AAGGGCGGCCGAACATACG | Rev primer to validate pRL100027 deletion mutant | This work |
| oxp3644 | CGCGCGCAAAGTCAAACG | Fwd primer to amplify internal fragment of pRL100027 – negative mutant mapping control | This work |
| oxp3645 | GCTGGGCGCCTATTTCACG | Rev primer to amplify internal fragment of pRL100027 – negative mutant mapping control | This work |
| oxp3666 | ACGCCGCCCATACTTACG | Fwd primer to validate RL4638 deletion mutant | This work |
| oxp3667 | GGTCTGCCACGCCATTTGC | Rev primer to validate RL4638 deletion mutant | This work |
| oxp3668 | TGACCGCATTGCCGTTGG | Fwd primer to amplify internal fragment of RL4638 – negative mutant mapping control | This work |
| oxp3669 | CGGCAATAAGGTCGCAGCG | Rev primer to amplify internal fragment of RL4638 – negative mutant mapping control | This work |
| oxp3769 | CGAATTCGAGCTCGGTACCCGATAAAGAACAGCAGGATCACATCG | Fwd primer for amplification of RL0685 upstream flanking region for mutagenesis | This work |
| oxp3770 | ATCCGATAGGCGCCTGTCCCCAACCACTTTC | Rev primer for amplification of RL0685 upstream flanking region for mutagenesis | This work |
| oxp3771 | GGGACAGGCGCCTATCGGATGATTGCCTC | Fwd primer for amplification of RL0685 downstream flanking region for mutagenesis | This work |
| oxp3772 | GTCGACTCTAGAGGATCCCCCCGACTTCAGCATCACCTTC | Rev primer for amplification of RL0685 downstream flanking region for mutagenesis | This work |
| oxp4021 | CCATCTCATCCCTGCGTGTCTCCGACTCA GCTAAGGTAACGATGATGTCCACG  AGGTCTCT | IT_BarSeq_1 (barcode underlined) | This work |
| oxp4022 | CCATCTCATCCCTGCGTGTCTCCGACTCAGTAAGGAGAACGATGATGTCCACGAGGTCTCT | IT_BarSeq_2 | This work |
| oxp4023 | CCATCTCATCCCTGCGTGTCTCCGACTCAGAAGAGGATTCGATGATGTCCACGAGGTCTCT | IT_BarSeq_3 | This work |
| oxp4024 | CCATCTCATCCCTGCGTGTCTCCGACTCAGTACCAAGATCGATGATGTCCACGAGGTCTCT | IT_BarSeq_4 | This work |
| oxp4025 | CCATCTCATCCCTGCGTGTCTCCGACTCAGCAGAAGGAACGATGATGTCCACGAGGTCTCT | IT_BarSeq_5 | This work |
| oxp4026 | CCATCTCATCCCTGCGTGTCTCCGACTCAGCTGCAAGTTCGATGATGTCCACGAGGTCTCT | IT_BarSeq_6 | This work |
| oxp4027 | CCTCTCTATGGGCAGTCGGTGATGTCGACCTGCAGCGTACG | IT_BarSeq_3’specific extension | This work |
| oxp4057 | CGGAGCTTGCGTGTACCTTG | Fwd primer to validate RL0685 deletion mutant | This work |
| oxp4058 | TCTCGGCGGACATCAGAACC | Rev primer to validate RL0685 deletion mutant | This work |
| oxp4156 | CCATCTCATCCCTGCGTGTCTCCGACTCAGTTCGTGATTCGATGATGTCCACGAGGTCTCT | IT_BarSeq_7 | This work |
| oxp4157 | CCATCTCATCCCTGCGTGTCTCCGACTCAGTTCCGATAACGATGATGTCCACGAGGTCTCT | IT_BarSeq_8 | This work |
| oxp4158 | CCATCTCATCCCTGCGTGTCTCCGACTCAGTGAGCGGAACGATGATGTCCACGAGGTCTCT | IT_BarSeq_9 | This work |
| oxp4159 | CCATCTCATCCCTGCGTGTCTCCGACTCAGCTGACCGAACGATGATGTCCACGAGGTCTCT | IT_BarSeq_10 | This work |
| oxp4160 | CCATCTCATCCCTGCGTGTCTCCGACTCAGTCCTCGAATCGATGATGTCCACGAGGTCTCT | IT_BarSeq_11 | This work |
| oxp4161 | CCATCTCATCCCTGCGTGTCTCCGACTCAGTAGGTGGTTCGATGATGTCCACGAGGTCTCT | IT_BarSeq_12 | This work |
| oxp4162 | CCATCTCATCCCTGCGTGTCTCCGACTCAGTCTAACGGACGATGATGTCCACGAGGTCTCT | IT_BarSeq_13 | This work |
| oxp4163 | CCATCTCATCCCTGCGTGTCTCCGACTCAGTTGGAGTGTCGATGATGTCCACGAGGTCTCT | IT_BarSeq_14 | This work |
| oxp4164 | CCATCTCATCCCTGCGTGTCTCCGACTCAGTCTAGAGGTCGATGATGTCCACGAGGTCTCT | IT_BarSeq_15 | This work |
| oxp4165 | CCATCTCATCCCTGCGTGTCTCCGACTCAGTCTGGATGACGATGATGTCCACGAGGTCTCT | IT_BarSeq_16 | This work |
| p653 | CCTGTTCGACCGTCTTGATGG | Downstream primer to validate RL4085 Tn5 mutant, used with Tn5 primer | (2) |
| p654 | CTCCGTCGCCTGGGTGATGGT | Upstream primer to validate RL4085 Tn5 mutant, used with Tn5 primer | (2) |
| p1369 | CTTCGTGATCCGTTCGGCGT | Primer to validate RL4705 (*leuD*) pK19 mutant | This work |
| p1518 | TGATTACGCCAAGCTGATCGGACTTGTGCTTGTC | Fwd primer for amplification of RL0033 (*manX*) internal | This work |
| p1519 | GCAGGCATGCAAGCTACCATCTGCACGAATTTGG | Rev primer for amplification of RL0033 (*manX*) internal | This work |
| p1539 | TGATTACGCCAAGCTGCGGTTCTTCAAGCAGTTCG | Fwd primer for amplification of RL1440 (*degP1*) internal | This work |
| p1540 | GCAGGCATGCAAGCTTTGATCGTCTCACCGTTCAG | Rev primer for amplification of RL1440 (*degP1*) internal | This work |
| p1541 | GAGCATTTCTGGCCGCATCC | Upstream primer to validate RL1440 (*degP1*) pK19 mutant | This work |
| P1542 | CGATGACGTCGTAGGTAT | Downstream primer to validate RL1440 (*degP1*) pK19 mutant | This work |
| p1588 | ACTAGTGAAAGCCGAAGCAAGCAGCC | Fwd primer for amplification of RL4705 (*leuD*) internal | This work |
| p1589 | ACTAGTTCGCGCCTCTCCACCTCGAC | Rev primer for amplification of RL4705 (*leuD*) internal | This work |
| p1786 | CGAATATCAGGCCAAGGTC | Upstream primer for validation of RL2096 Tn5 mutant, used with Tn5 primer | This work |
| p1789 | AAGACGTCGATATCGC | Downstream primer for validation of RL2096 Tn5 mutant, used with Tn5 primer | This work |
| pr1498 | ATCATCAAGCCCATGATGGA | Fwd primer to validate RL0033 (*manX*) pK19 mutant | (13) |
| pr1499 | CCTTCCGGATTGGTCAATCT | Rev primer to validate RL0033 (*manX*) pK19 mutant | (13) |

**SI Appendix, Table 5. Mutant phenotypes for growth in pea rhizosphere.**

| **Mutant** | **mCherry events/ g root** | **GFP events/ g root** | **% of WT** | **Standard deviation** | **Paired *t* test**  **(*p-*value)** |
| --- | --- | --- | --- | --- | --- |
| **OPS2453 (WT Tn7 mCherry) v OPS2452 (WT Tn7 GFP)** | | | | | |
| A | 4.75E+07 | 5.05E+07 | 106.25 | 5.02 | 0.148939813 |
| B | 3.68E+07 | 3.96E+07 | 107.69231 |  |  |
| C | 1.59E+08 | 1.86E+08 | 116.94915 |  |  |
| D | 1.58E+08 | 1.69E+08 | 107.01754 |  |  |
| **OPS3675 (Δ*nifH* Tn7 mCherry) v OPS3677 (Δ*purC1* Tn7 GFP)** | | | | | |
| A | 5.89E+05 | 4.54E+05 | 77.07 | 3.29 | 0.132523 |
| B | 2.87E+06 | 2.39E+06 | 83.52 |  |  |
| C | 8.34E+05 | 6.74E+05 | 80.85 |  |  |
| D | 9.88E+04 | 7.57E+04 | 76.56 |  |  |
| **OPS3675 (Δ*nifH* Tn7 mCherry) v OPS3670 (Δ*leuD* Tn7 GFP)** | | | | | |
| A | 4.97E+05 | 4.40E+04 | 8.86 | 2.93 | 0.031531 |
| B | 2.30E+06 | 1.20E+05 | 5.20 |  |  |
| C | 6.32E+04 | ND | - |  |  |
| D | 1.33E+06 | 3.68E+04 | 2.77 |  |  |
| E | 1.59E+06 | 4.05E+04 | 2.54 |  |  |
| **OPS3675 (Δ*nifH* Tn7 mCherry) v OPS3673 (Δ*gltB* Tn7 GFP)** | | | | | |
| A | 9.95E+05 | 1.72E+04 | 1.73 | 8.79 | 0.050764 |
| B | 5.36E+05 | 1.17E+05 | 21.91 |  |  |
| C | 2.08E+06 | 1.29E+05 | 6.21 |  |  |
| D | 2.45E+06 | 4.82E+04 | 1.97 |  |  |
| E | 4.92E+06 | 5.75E+04 | 1.17 |  |  |
| **OPS3675 (Δ*nifH* Tn7 mCherry) v OPS3678 (Δ*eryB* Tn7 GFP)** | | | | | |
| A | 2.53E+06 | 9.05E+04 | 3.58 | 1.85 | 0.017812 |
| B | 3.49E+06 | 1.58E+05 | 4.52 |  |  |
| C | 3.37E+06 | 2.48E+05 | 7.35 |  |  |
| D | 1.05E+06 | 3.45E+04 | 3.28 |  |  |
| E | 5.46E+04 | ND | - |  |  |
| **OPS3675 (Δ*nifH* Tn7 mCherry) v OPS3666 (*degP1* Tn7 GFP)** | | | | | |
| A | 1.85E+06 | 1.17E+06 | 63.43 | 3.23 | 0.053652 |
| B | 9.61E+06 | 5.36E+06 | 55.79 |  |  |
| C | 9.19E+06 | 5.32E+06 | 57.86 |  |  |
| D | 4.28E+06 | 2.52E+06 | 58.86 |  |  |
| **OPS3675 (Δ*nifH* Tn7 mCherry) v OPS3667 (ΔRL0033 Tn7 GFP)** | | | | | |
| A | 1.15E+06 | 1.26E+06 | 109.68 | 22.65 | 0.918105 |
| B | 1.19E+06 | 1.44E+06 | 121.25 |  |  |
| C | 4.58E+05 | 2.99E+05 | 65.28 |  |  |
| D | 8.45E+06 | 8.23E+06 | 97.39 |  |  |
| E | 1.51E+05 | 1.19E+05 | 78.87 |  |  |
| **OPS3675 (Δ*nifH* Tn7 mCherry) v OPS3672 (ΔRL2096 Tn7 GFP)** | | | | | |
| A | 1.47E+06 | 1.89E+05 | 12.83 | 3.06 | 0.000481 |
| B | 9.45E+05 | 5.00E+04 | 5.29 |  |  |
| C | 8.82E+05 | 5.71E+04 | 6.48 |  |  |
| D | 1.41E+06 | 1.49E+05 | 10.56 |  |  |
| E | 9.86E+05 | 9.26E+04 | 9.39 |  |  |
| **OPS2453 (WT Tn7 mCherry) v OPS3698 (ΔpRL100027 Tn7 GFP)** | | | | | |
| A | 6.33E+07 | 2.04E+04 | 0.03 | 0.048 | 0.017991 |
| B | 7.93E+07 | 1.03E+05 | 0.13 |  |  |
| C | 1.43E+08 | 4.35E+04 | 0.03 |  |  |
| D | 6.73E+07 | 5.61E+04 | 0.08 |  |  |
| **OPS2453 (WT Tn7 mCherry) v OPS3700 (ΔRL4638 Tn7 GFP)** | | | | | |
| A | 3.08E+08 | 3.33E+06 | 1.08 | 0.43 | 0.014754 |
| B | 3.52E+08 | 4.69E+05 | 0.13 |  |  |
| C | 1.14E+08 | 4.23E+05 | 0.37 |  |  |
| D | 2.83E+08 | 7.20E+05 | 0.25 |  |  |
| **OPS3675 (Δ*nifH* Tn7 mCherry) v OPS3672 (Δ*pssD* Tn7 GFP)** | | | | | |
| A | 2.34E+06 | 5.11E+05 | 21.83 | 52.6 | 0.433482 |
| B | 2.38E+06 | 2.47E+06 | 104.16 |  |  |
| C | 3.33E+05 | 3.37E+05 | 101.10 |  |  |
| D | 1.12E+05 | 1.90E+05 | 170.23 |  |  |
| E | 4.87E+05 | 5.00E+05 | 102.67 |  |  |
| **OPS3675 (Δ*nifH* Tn7 mCherry) v OPS3669 (ΔRL3453 Tn7 GFP)** | | | | | |
| A | 4.19E+06 | 1.54E+05 | 3.67 | 1.06 | 0.051861 |
| B | 1.81E+05 | 2.80E+03 | 1.54 |  |  |
| C | 4.92E+06 | 1.91E+05 | 3.88 |  |  |
| D | 4.77E+06 | 1.56E+05 | 3.27 |  |  |
| E | 1.65E+05 | ND | - |  |  |
| **OPS3675 (Δ*nifH* Tn7 mCherry) v OPS3668 (ΔpRL120694 Tn7 GFP)** | | | | | |
| A | 1.14E+06 | 7.89E+05 | 69.32 | 11.63 | 0.0494 |
| B | 3.16E+05 | 1.56E+05 | 49.40 |  |  |
| C | 1.24E+06 | 6.86E+05 | 55.42 |  |  |
| D | 2.92E+05 | 1.56E+05 | 53.38 |  |  |
| E | 4.28E+06 | 3.29E+06 | 76.75 |  |  |
| **OPS3675 (Δ*nifH* Tn7 mCherry) v OPS3663 (ΔpRL120291 Tn7 GFP)** | | | | | |
| A | 2.58E+06 | 5.04E+05 | 19.51 | 3.82 | 0.0889 |
| B | 7.69E+05 | 2.17E+05 | 28.17 |  |  |
| C | 1.05E+07 | 1.93E+06 | 18.45 |  |  |
| D | 6.83E+06 | 1.41E+06 | 20.69 |  |  |
| E | 8.74E+05 | 1.90E+05 | 21.77 |  |  |
| **OPS3675 (Δ*nifH* Tn7 mCherry) v OPS3665 (Δ*glnII* Tn7 GFP)** | | | | | |
| A | 8.87E+06 | 2.12E+06 | 23.93 | 22.6 | 0.3073 |
| B | 2.37E+05 | 1.67E+05 | 70.44 |  |  |
| C | 6.13E+05 | 4.07E+05 | 66.31 |  |  |
| D | 2.92E+05 | 2.24E+05 | 76.91 |  |  |
| E | 2.63E+06 | 2.08E+06 | 79.03 |  |  |
| **OPS3675 (Δ*nifH* Tn7 mCherry) v OPS3676 (ΔRL4382 Tn7 GFP)** | | | | | |
| A | 2.02E+06 | 3.99E+05 | 19.77 | 4.9 | 0.028734 |
| B | 3.79E+06 | 5.97E+05 | 15.75 |  |  |
| C | 1.72E+06 | 4.10E+05 | 23.91 |  |  |
| D | 2.24E+06 | 5.12E+05 | 22.81 |  |  |
| E | 2.69E+05 | 7.78E+04 | 28.91 |  |  |
| **OPS3675 (Δ*nifH* Tn7 mCherry) v OPS3662 (Δ*rem* Tn7 GFP)** | | | | | |
| A | 1.45E+06 | 9.65E+05 | 66.41 | 24.79712873 | 0.148592 |
| B | 1.23E+07 | 7.35E+06 | 59.60 |  |  |
| C | 2.10E+06 | 1.84E+06 | 87.59 |  |  |
| D | 6.23E+05 | 5.72E+05 | 91.86 |  |  |
| E | 3.86E+06 | 1.16E+06 | 30.08 |  |  |
| **OPS2453 (WT Tn7 mCherry) v OPS3701 (Δ*icpA* Tn7 GFP)** | | | | | |
| A | 3.10E+07 | 2.43E+07 | 78.33 | 21.07261 | 0.13 |
| B | 8.88E+07 | 6.12E+07 | 68.97 |  |  |
| C | 7.04E+07 | 3.98E+07 | 56.52 |  |  |
| D | 5.00E+07 | 5.00E+07 | 100.00 |  |  |
| E | 6.49E+06 | 6.94E+06 | 106.90 |  |  |

**SI Appendix, Table 6. Mutant phenotypes for colonisation of pea roots.**

| **Mutant** | **mCherry events/ g root** | **GFP events/ g root** | **% of WT** | **Standard deviation** | **Paired *t* test**  **(*p-*value)** |
| --- | --- | --- | --- | --- | --- |
| **OPS2453 (WT Tn7 mCherry) v OPS2452 (WT Tn7 GFP)** | | | | | |
| A | 6.47E+05 | 7.12E+05 | 110.11 | 13.48 | 0.148939813 |
| B | 8.18E+05 | 1.02E+06 | 124.24 |  |  |
| C | 4.16E+05 | 4.94E+05 | 118.85 |  |  |
| D | 3.22E+05 | 3.00E+05 | 93.42 |  |  |
| **OPS3675 (Δ*nifH* Tn7 mCherry) v OPS3677 (Δ*purC1* Tn7 GFP)** | | | | | |
| A | 5.73E+05 | 3.97E+05 | 69.18 | 6.05 | 0.016657 |
| B | 7.63E+05 | 4.72E+05 | 61.93 |  |  |
| C | 3.18E+05 | 2.02E+05 | 63.41 |  |  |
| D | 1.03E+06 | 5.94E+05 | 57.50 |  |  |
| E | 5.42E+05 | 3.95E+05 | 72.78 |  |  |
| **OPS3675 (Δ*nifH* Tn7 mCherry) v OPS3670 (Δ*leuD* Tn7 GFP)** | | | | | |
| A | 8.40E+05 | 6.05E+04 | 7.20 | 4.11 | 0.12022 |
| B | 1.19E+06 | ND | - |  |  |
| C | 4.59E+05 | 7.19E+02 | 0.16 |  |  |
| D | 1.71E+06 | 8.12E+01 | 0.00 |  |  |
| E | 1.35E+06 | ND | - |  |  |
| **OPS3675 (Δ*nifH* Tn7 mCherry) v OPS3673 (Δ*gltB* Tn7 GFP)** | | | | | |
| A | 1.20E+06 | 1.62E+04 | 1.35 | 0.22 | 0.068983 |
| B | 3.51E+06 | 4.67E+04 | 1.33 |  |  |
| C | 1.43E+06 | 2.43E+04 | 1.71 |  |  |
| D | 7.26E+05 | 1.25E+04 | 1.72 |  |  |
| E | 4.76E+05 | ND | - |  |  |
| **OPS3675 (Δ*nifH* Tn7 mCherry) v OPS3678 (Δ*eryB* Tn7 GFP)** | | | | | |
| A | 1.58E+06 | 3.15E+04 | 1.99 | 2.04 | 0.007459 |
| B | 1.13E+06 | 4.81E+04 | 4.28 |  |  |
| C | 1.35E+06 | 4.95E+04 | 3.66 |  |  |
| D | 1.15E+06 | 2.53E+04 | 2.21 |  |  |
| E | 3.03E+05 | 2.14E+04 | 7.05 |  |  |
| **OPS3675 (Δ*nifH* Tn7 mCherry) v OPS3666 (*degP1* Tn7 GFP)** | | | | | |
| A | 1.60E+06 | 5.41E+05 | 33.74 | 17.53 | 0.054948 |
| B | 1.01E+06 | 1.43E+05 | 14.15 |  |  |
| C | 2.63E+05 | 1.41E+05 | 53.68 |  |  |
| D | 6.83E+05 | 2.01E+05 | 29.34 |  |  |
| **OPS3675 (Δ*nifH* Tn7 mCherry) v OPS3667 (ΔRL0033 Tn7 GFP)** | | | | | |
| A | 8.30E+05 | 4.51E+05 | 54.32 | 8.25 | 0.006947 |
| B | 4.50E+05 | 2.75E+05 | 61.06 |  |  |
| C | 6.08E+05 | 4.25E+05 | 69.89 |  |  |
| D | 6.45E+05 | 4.54E+05 | 70.39 |  |  |
| E | 5.70E+05 | 4.26E+05 | 74.79 |  |  |
| **OPS3675 (Δ*nifH* Tn7 mCherry) v OPS3672 (ΔRL2096 Tn7 GFP)** | | | | | |
| A | 1.17E+06 | 1.53E+05 | 13.12 | 3.24 | 0.000624 |
| B | 7.27E+05 | 3.73E+04 | 5.13 |  |  |
| C | 6.75E+05 | 4.32E+04 | 6.40 |  |  |
| D | 1.12E+06 | 1.20E+05 | 10.74 |  |  |
| E | 7.62E+05 | 7.28E+04 | 9.56 |  |  |
| **OPS2453 (WT Tn7 mCherry) v OPS3698 (ΔpRL100027 Tn7 GFP)** | | | | | |
| A | 6.85E+05 | 1.21E+04 | 1.77 | 0.73 | 0.012085 |
| B | 1.79E+06 | 1.07E+04 | 0.60 |  |  |
| C | 3.07E+06 | 3.56E+03 | 0.12 |  |  |
| D | 1.28E+06 | 1.72E+04 | 1.35 |  |  |
| E | 7.19E+05 | 1.46E+04 | 2.02 |  |  |
| F | 1.05E+06 | 1.62E+04 | 1.55 |  |  |
| G | 4.63E+05 | ND | - |  |  |
| H | 6.85E+05 | 1.21E+04 | 1.77 |  |  |
| **OPS2453 (WT Tn7 mCherry) v OPS3700 (ΔRL4638 Tn7 GFP)** | | | | | |
| A | 8.72E+05 | ND | - | 0.76 | 0.016265 |
| B | 1.24E+06 | 3.84E+03 | 0.31 |  |  |
| C | 1.95E+06 | 3.46E+03 | 0.18 |  |  |
| D | 2.62E+05 | 1.42E+03 | 0.54 |  |  |
| E | 3.44E+05 | ND | - |  |  |
| F | 1.14E+06 | ND | - |  |  |
| G | 8.38E+05 | 1.66E+04 | 1.98 |  |  |
| H | 1.51E+06 | 2.56E+03 | 0.17 |  |  |
| **OPS3675 (Δ*nifH* Tn7 mCherry) v OPS3672 (Δ*pssD* Tn7 GFP)** | | | | | |
| A | 8.64E+05 | 1.04E+06 | 120.52 | 10.40 | 0.010246 |
| B | 4.88E+05 | 6.81E+05 | 139.47 |  |  |
| C | 3.98E+05 | 4.52E+05 | 113.65 |  |  |
| D | 4.55E+05 | 5.83E+05 | 128.04 |  |  |
| E | 4.50E+05 | 5.24E+05 | 116.34 |  |  |
| **OPS3675 (Δ*nifH* Tn7 mCherry) v OPS3669 (ΔRL3453 Tn7 GFP)** | | | | | |
| A | 3.24E+06 | 3.98E+04 | 1.23 | 0.38 | 0.011337 |
| B | 1.87E+06 | 1.98E+04 | 1.06 |  |  |
| C | 1.87E+06 | 3.18E+04 | 1.70 |  |  |
| D | 1.25E+06 | 1.13E+04 | 0.90 |  |  |
| E | 8.31E+05 | 1.45E+04 | 1.75 |  |  |
| **OPS3675 (Δ*nifH* Tn7 mCherry) v OPS3668 (ΔpRL120694 Tn7 GFP)** | | | | | |
| A | 3.01E+05 | 1.49E+05 | 49.51 | 10.46 | 0.009190 |
| B | 1.28E+06 | 5.23E+05 | 40.96 |  |  |
| C | 1.61E+06 | 7.43E+05 | 46.16 |  |  |
| D | 6.90E+05 | 1.85E+05 | 26.79 |  |  |
| E | 1.69E+06 | 9.11E+05 | 53.94 |  |  |
| **OPS3675 (Δ*nifH* Tn7 mCherry) v OPS3663 (ΔpRL120291 Tn7 GFP)** | | | | | |
| A | 6.33E+05 | 6.01E+04 | 9.50 | 1.24 | 0.000231 |
| B | 6.08E+05 | 6.67E+04 | 10.97 |  |  |
| C | 4.14E+05 | 4.20E+04 | 10.13 |  |  |
| D | 5.33E+05 | 4.83E+04 | 9.05 |  |  |
| E | 4.67E+05 | 5.69E+04 | 12.17 |  |  |
| **OPS3675 (Δ*nifH* Tn7 mCherry) v OPS3665 (Δ*glnII* Tn7 GFP)** | | | | | |
| A | 3.35E+05 | 3.14E+04 | 9.39 | 20.35 | 0.001941 |
| B | 4.91E+05 | 1.89E+05 | 38.45 |  |  |
| C | 6.14E+05 | 1.06E+05 | 17.32 |  |  |
| D | 5.63E+05 | 3.19E+05 | 56.75 |  |  |
| E | 5.83E+05 | 2.88E+05 | 49.39 |  |  |
| **OPS3675 (Δ*nifH* Tn7 mCherry) v OPS3676 (ΔRL4382 Tn7 GFP)** | | | | | |
| A | 5.06E+05 | 3.89E+04 | 7.68 | 3.11 | 0.011234 |
| B | 8.87E+05 | 2.69E+04 | 3.03 |  |  |
| C | 8.69E+05 | 9.56E+04 | 11.00 |  |  |
| D | 1.82E+06 | 1.82E+05 | 9.96 |  |  |
| E | 7.48E+05 | 5.01E+04 | 6.70 |  |  |
| **OPS3675 (Δ*nifH* Tn7 mCherry) v OPS3662 (Δ*rem* Tn7 GFP)** | | | | | |
| A | 6.35E+05 | 2.56E+05 | 40.40 | 16.95 | 0.00678 |
| B | 1.41E+06 | 3.68E+04 | 2.61 |  |  |
| C | 1.20E+06 | 4.99E+05 | 41.64 |  |  |
| D | 1.36E+06 | 2.00E+05 | 14.72 |  |  |
| E | 1.60E+06 | 3.02E+05 | 18.92 |  |  |
| **OPS2453 (WT Tn7 mCherry) v OPS3701 (Δ*icpA* Tn7 GFP)** | | | | | |
| A | 2.31E+06 | 8.11E+05 | 35.15 | 12.71 | 0.024607 |
| B | 1.23E+06 | 5.25E+05 | 42.59 |  |  |
| C | 1.01E+06 | 3.23E+05 | 31.87 |  |  |
| D | 4.70E+05 | 1.99E+05 | 42.39 |  |  |
| E | 4.19E+04 | 3.06E+04 | 72.94 |  |  |
| F | 3.64E+05 | 1.73E+05 | 47.51 |  |  |
| G | 3.74E+06 | 1.41E+06 | 37.78 |  |  |
| H | 9.00E+05 | 3.64E+05 | 40.45 |  |  |

**Methods (additional information)**

**Seed sterilisation and growth.** *Pisum* *sativum* L. (Pea) seeds were surface sterilised by immersion in 70% ethanol for 30 s, followed by 2.8% sodium hypochlorite for 5 min, and rinsed 6 times with sterile dH_2_O. *Lens culinaris* Medik (Lentil) seeds were surface sterilized by immersion in 0.56% sodium hypochlorite for 5 min and washed 6 times with sterile dH_2_O. *Lathyrus latifolius* L. (*Lathyrus*) seeds were submerged for 8 min in sulfuric acid and washed 6 times with sterile dH_2_O. *Medicago sativa* L. (Alfalfa) seeds were immersed in 95% ethanol for 45 min, followed by 5.25% bleach for 15 min, and rinsed 6 times with sterile dH_2_O. *Hordeum vulgare* L. (Barley) seeds were surface sterilised in 70% ethanol for 2 min, 7% sodium hypochlorite + 0.1% Tween20 (Sigma Aldrich) for 5 min and rinsed 6 times with sterile dH_2_O. Sterile seeds were placed directly into sterilised boiling tubes to germinate in a controlled growth chamber set at 23°C with a 16 h light 8 h dark photoperiod.

**Quantification of bacterial populations by flow cytometry.** Flow rates were set to low speed/high sensitivity (3.66 µL/min) and 10µl were counted for each sample. Using Cellstream® Analysis 1.3.384 software, the Bacteria population was defined as the concentrated events area when plotting size (FSC) and granularity (SSC). The Bacteria population was then gated based on FSC (threshold > 0) and the aspect-ratio of SSC (threshold >0.4) defining the Singlets population. Singlets events were gated based on their fluorescence emission, generating two Colour populations: Red and Yellow, for mCherry and sfGFP respectively*.* The Red population are Singlets events detected in the 561 - 611/31 channel above 3000 fluorescence intensity (FI) units. The Yellow population are Singlets events detected 488 - 528/46 channel above 3000 FI units. Afterwards, we created two Combined populations defined as presence/absence of Red and Yellow: R population (exclusively Red), Y (exclusively Yellow). The number of events/mL^-1^ was recorded for each Combined population in each sample and transformed into events/g root^-1^.

**References**

1. Johnston AWB, Beringer JE. Identification of the *Rhizobium* strains in pea root nodules using genetic markers. Microbiology. 1975;87(2):343-50.

2. Karunakaran R, Ramachandran VK, Seaman JC, East AK, Mouhsine B, Mauchline TH, et al. Transcriptomic analysis of *Rhizobium leguminosarum* biovar *viciae* in symbiosis with host plants *Pisum sativum* and *Vicia cracca*. J Bacteriol. 2009;191(12):4002-14.

3. Ramachandran VK, East AK, Karunakaran R, Downie JA, Poole PS. Adaptation of *Rhizobium leguminosarum* to pea, alfalfa and sugar beet rhizospheres investigated by comparative transcriptomics. Genome Biol. 2011;12(10):R106.

4. Wheatley RM, Ford BL, Li L, Aroney STN, Knights HE, Ledermann R, et al. Lifestyle adaptations of *Rhizobium* from rhizosphere to symbiosis. Proc Natl Acad Sci U S A. 2020;117(38):23823-34.

5. Parsons J, D. , Cocker C, R. , East A, K., Wheatley R, M., Ramachandran V, K., Kaschani F, et al. Factors governing attachment of *Rhizobium leguminosarum* to legume roots at acid, neutral and alkaline pHs. Under review. 2024.

6. Jorrin B, Haskett TL, Knights HE, Martyn A, Dolliver J, Ledermann R, et al. Stable, fluorescent markers for tracking synthetic communities and assembly dynamics. Microbiome. 2024; Accepted.

7. Perry BJ, Yost CK. Construction of a *mariner*-based transposon vector for use in insertion sequence mutagenesis in selected members of the *Rhizobiaceae*. BMC Microbiol. 2014;14(1):298.

8. Ditta G, Stanfield S, Corbin D, Helinski DR. Broad host range DNA cloning system for gram-negative bacteria: construction of a gene bank of *Rhizobium meliloti*. Proc Natl Acad Sci U S A. 1980;77(12):7347-51.

9. Schäfer A, Tauch A, Jäger W, Kalinowski J, Thierbach G, Pühler A. Small mobilizable multi-purpose cloning vectors derived from the *Escherichia coli* plasmids pK18 and pK19: selection of defined deletions in the chromosome of *Corynebacterium glutamicum*. Gene. 1994;145(1):69-73.

10. Poole PS, Schofiel NA, Reid CJ, Drew EM, Walshaw DL. Identification of chromosomal genes located downstream of *dctD* that affect the requirement for calcium and the lipopolysaccharide layer of *Rhizobium leguminosarum*. Microbiology 1994;140:2797–809.

11. Goodman AL, McNulty NP, Zhao Y, Leip D, Mitra RD, Lozupone CA, et al. Identifying genetic determinants needed to establish a human gut symbiont in its habitat. Cell Host Microbe. 2009;6(3):279-89.

12. Williams A, Wilkinson A, Krehenbrink M, Russo DM, Zorreguieta A, Downie JA. Glucomannan-mediated attachment of *Rhizobium leguminosarum* to pea root hairs is required for competitive nodule infection. J Bacteriol. 2008;190(13):4706-15.

13. Sánchez-Cañizares C, Prell J, Pini F, Rutten P, Kraxner K, Wynands B, et al. Global control of bacterial nitrogen and carbon metabolism by a PTSNtr-regulated switch. Proc Natl Acad Sci U S A. 2020;117(19):10234-45.
